# Opposite-view digital holographic microscopy with autofocusing capability

**DOI:** 10.1038/s41598-017-04568-x

**Published:** 2017-06-26

**Authors:** Juanjuan Zheng, Peng Gao, Xiaopeng Shao

**Affiliations:** 1 School of Physics and Optoelectronic Engineering, Xidian University, Xi’an, 710071 China; 2 Institute of Applied Physics, Karlsruhe Institute of Technology, 76128 Karlsruhe, Germany; 3 Institute of Nanotechnology, Karlsruhe Institute of Technology, 76344 Eggenstein-Leopoldshafen, Germany

## Abstract

Digital holographic microscopy (DHM) has its intrinsic ability to refocusing a sample by numerically propagating an object wave from its hologram plane to its image plane. In this paper opposite-view digital holographic microscopy (OV-DHM) is demonstrated for autofocusing, namely, digitally determining the location of the image plane, and refocusing the object wave without human intervention. In OV-DHM, a specimen is illuminated from two sides in a 4π-alike configuration, and two holograms are generated and recorded by a CCD camera along two orthogonal polarization orientations. The image plane of the sample is determined by finding the minimal variation between the two object waves, and consequently refocusing is performed by propagating the waves to the image plane. Furthermore, the field of view (FOV) of OV-DHM can be extended by combining the two object waves which have an angle in-between. The proposed technique also has the potential to reduce speckle noise and out-of-focus background.

## Introduction

Digital holographic microscopy (DHM) is a non-invasive, high-resolution, whole-field technique for measuring microscopic specimens, especially translucent sample.^1–5^ In microcopy, samples are often observed in in-focus scene through manual or mechanical focusing. However, this focusing manner becomes nearly impossible when measuring a moving sample or dynamic processes. In DHM, the difficulty in mechanical focusing is circumvented by a refocusing process: propagating an object wave from hologram plane to image plane. Notably, the DHM enables to refocus laterally-separated regions of a hologram to different focal planes and consequently, it can provide 3D information of the sample.^6^ A key issue in reconstructing a refocused image from the out-of-focus hologram is the image plane determination, i.e., to find the distance between the hologram plane and the image plane. Hitherto, there have been many reports on image plane detection, which are based on amplitude analysis,^7–9^ intensity gradient,^10^ self-entropy,^11^ local intensity variance,^12^ spectral norms,^13^ wavelet theory,^14^ and so on.^15–20^ Recently, we also reported non-conventional illumination based image plane determination approaches, which are based on two-wavelength illumination,^21^ off-axis illuminations^22^ or structured illumination.^23^ The image plane was determined by finding the minimal difference between the reconstructed object waves which are aligned with two wavelength illuminations, two off-axis illuminations, or two diffraction orders of structured illumination.

In aside of the image plane determination, the non-conventional illumination schemes^21–26^ enable an increased data acquisition along the designed illuminations. Normally, DHM uses a plane wave for illumination and consequently, its resolution and axial sectioning ability of DHM is worse than that of the conventional microscope, which employs Koehler illumination with a boarder spectrum. Off-axis illumination,^22, 24^ structured illumination^23^ and speckle illumination^25, 26^ can improve the lateral resolution of DHM, and in the meantime contribute to improving the axial sectioning ability of DHM. Recently, opposed-view dark-field digital holographic microscopy was proposed, which collects the scattered light concurrently from both opposite views, and therefore improves the contrast of internal structures and as well the signal-to-noise ratio.^27, 28^

In this paper, we present an opposite-view digital holographic microscopy (OV-DHM) for autofocusing and field of view (FOV) extension. The OV-DHM enables to determine the image plane automatically and refocus a sample digitally, providing the possibility to image moving samples or dynamic processes. Compared with conventional autofocusing methods, the presented technique can be used for more general samples. Furthermore, OV-DHM can extend the field of view (FOV) of imaging by combining the two reconstructed object waves, which have an angle in-between. Furthermore, OV-DHM can collect more frequency spectrum (from two sides), and thus it has the potential to suppress out-of-focus background.

## Results

### Configuration of OV-DHM

**Figure 1:**
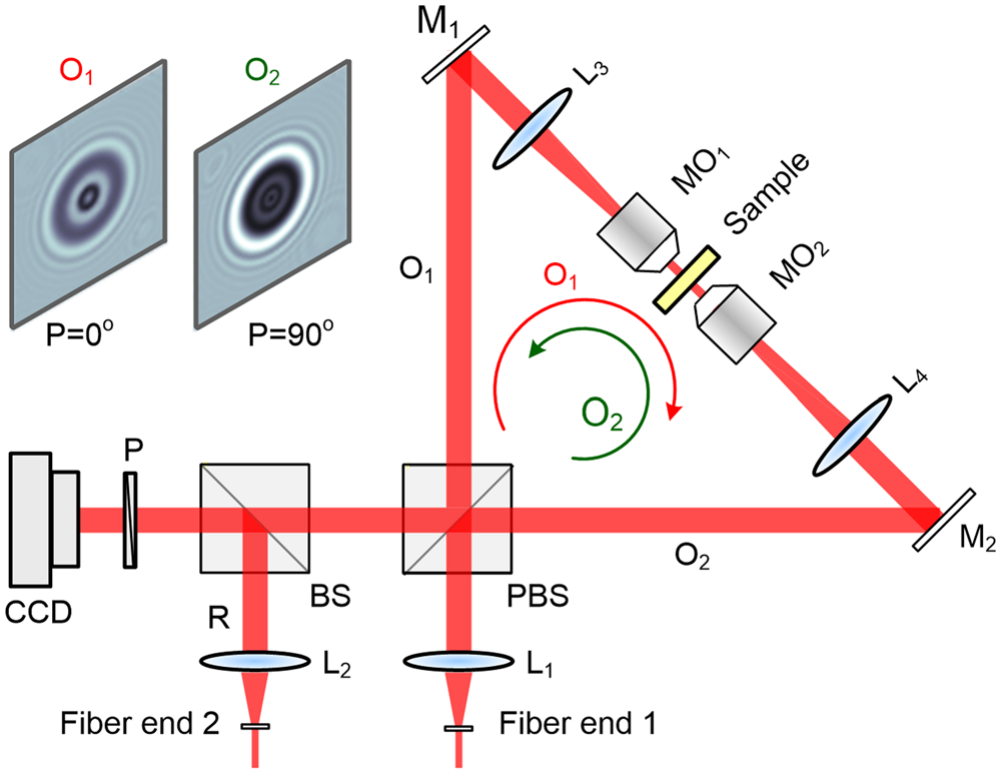
Experimental setup of opposite-view digital holographic microscopy (OV-DHM). MO_1_ and MO_2_, microscopic objectives; L_1_–L_4_, achromatic lenses; BS, beamsplitter; PBS, polarization-maintaining beamsplitter; M_1_ and M_2_, mirrors; P, polarizer; CCD, Charge-coupled device; O_1_ and O_2_, Object waves linearly polarized along horizontal (0°) and vertical (90°) directions, respectively; R, reference wave linearly polarized at an azimuth of 45°.

The schematic diagram of our home-built opposite-view digital holographic microscopy (OV-DHM) is shown in Fig. 1. The experiment setup is based on a common-path Sagnac configuration, which is comprised of a polarization-maintaining beamsplitter PBS and two mirrors M_1_ and M_2_. A laser beam from the fiber end 1 is split by the PBS into two copies, of which the polarizations are along the horizontal and vertical directions, respectively. The copy which has horizontal polarization goes through the Sagnac configuration clockwise, while the other one goes through the Sagnac configuration anti-clockwise. Two telescope systems MO_1_-L_3_ and L_4_-MO_2_ are placed between M_1_ and M_2_, and are used to image a sample with a magnification of 20X. A sample is located between the objectives MO_1_ and MO_2_. After the illumination beams transmit the sample in opposite directions, the output object waves (namely *O*_1_ and *O*_2_) are magnified by the two telescope systems, and superimposed with a common reference wave *R* via a non-polarizing beamsplitter BS. The reference wave is linearly polarized with its polarization azimuth 45° with respect to the polarizations of *O*_1_ and *O*_2_. Two hologram *I*_1_ = |*O*_1_ + *R*|^2^ and *I*_2_ = |*O*_2_ + *R*|^2^ were obtained separately by rotating the polarizer *P* to horizontal and vertical directions, respectively. We note that the reference wave *R* was adjusted to have an angle of 10 ± 0.1 mrad with respect to the two in-line object waves. It is worthy to mention that the OV-DHM configuration can be further upgraded by employing two CCD cameras to record the two opposite-view holograms at the same time (see Supplementary Fig. 1).

### Autofocusing principle of the OV-DHM

**Figure 2:**
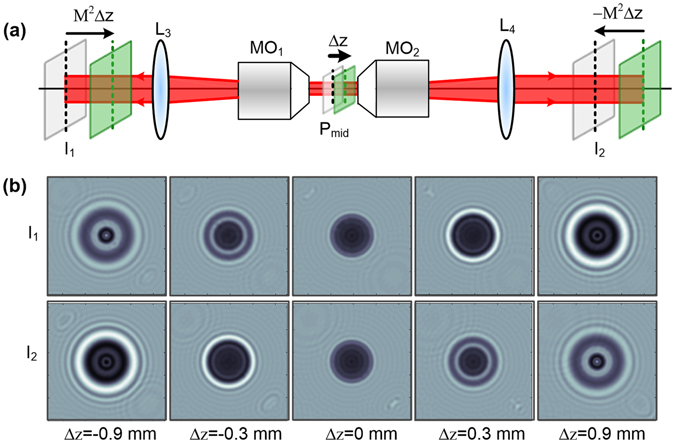
Schematics of autofocusing of OV-DHM. (**a**) Illumination and imaging schematics of OV-DHM; P_mid_ denotes the middle plane of telescope system MO_1_-L_3_ and MO_2_-L_4_; M denotes the magnification of the two telescope systems. (**b**) The simulated images of a sample on CCD plane when it moves from left to right with Δz = −0.9 mm, −0.3 mm, 0 mm, 0.3 mm and 0.9mm from the middle plane P_mid_.

As seen in Fig. 2(a), a CCD is placed to record the in-focus images of the middle plane P_mid_ of the two objectives clockwise and anti-clockwise in OV-DHM. If a sample is located to have a distance Δ*z* from P_mid_, the hologram *I*_1_ will have a defocusing distance *M* ^2^Δ*z* along clockwise direction, while the hologram *I*_2_ will have an opposite defocusing distance −*M* ^2^Δ*z* along the anti-clockwise direction. Here *M* is the magnification of the OV-DHM system. For reconstruction, the two object waves, *O*_r1_ and *O*_r2_, which have an arbitrary distance −Δ*d* from *I*_1_ (and Δ*d* from *I*_2_) can be reconstructed by using angular spectrum method:^29^

1 $$\{\begin{array}{c}{O}_{r1}=F{T}^{-1}\{FT\{{I}_{1}{R}_{D}\}\cdot {W}_{filter}\cdot \exp [-ik\Delta d\sqrt{1-{(\lambda \xi )}^{2}-{(\lambda \eta )}^{2}}]\},\\ {O}_{r2}=F{T}^{-1}\{FT\{{I}_{2}{R}_{D}\}\cdot {W}_{filter}\cdot \exp [ik\Delta d\sqrt{1-{(\lambda \xi )}^{2}-{(\lambda \eta )}^{2}}]\}.\end{array}$$

Here *k* = 2π/λ denotes the wave vector; *FT*{·} and *FT* ^−1^{·} denote Fourier-transformation and inverse Fourier-transformation operators, *ξ* and *η* are the spatial coordinates in the frequency domain. *R*_D_ is a digitalized reference waves, which has a linear phase term (corresponding to the angle between object wave and reference wave) used to shift the spectrum of the real image to the center of the frequency domain. *W*_*filter*_(*ξ*, *η*) is the window function used to select the real images of *I*_1_ *R*_D_ and *I*_2_ *R*_D_ in the frequency domain, blocking their *dc* terms and twin images (see Supplementary Fig. S2). The cut-off frequency of *W*_*filter*_ was chosen to maintain the highest frequency of the object wave (defined with ω_0_). The angle between the object and reference waves should be designed to yield an off-axis hologram which has a carrier frequency $\ge$3ω_0_ in order to a separation between the real image, twin image and *dc* term.^30^ A CCD camera which has a sampling frequency $\ge$8ω_0_ is required to record the hologram.

OV-DHM has an intrinsic property that, the holograms *I*_1_ and *I*_2_ have opposite defocusing distances. Thus, the difference between |*O*_r1_(Δ*d*)|^2^ and |*O*_r2_(−Δ*d*)|^2^ reaches the minimum when the reconstruction distance Δ*d* is correct (match *M* ^2^Δz). Otherwise, the larger |Δ*d* − M^2^Δz| is, the larger is the variation of |*O*_r1_(Δ*d*)|^2^ − |*O*_r2_(−Δ*d*)|^2^. Thus, this property can be used as focus criterion to determine image plane of OV-DHM. For this purpose, the focus criterion can be defined as:

2 $$Cri({\rm{\Delta }}d)=RMS\{{|{O}_{r1}|}^{2}-{|{O}_{r2}|}^{2}\}$$

In Equation (2), *RMS* denotes the operation of root-mean-square operation. In implementation, an in-focus plane can be determined by finding the minimum of the criterion function in Equation (2).

### Autofocusing of lensless OV-DHM

**Figure 3:**
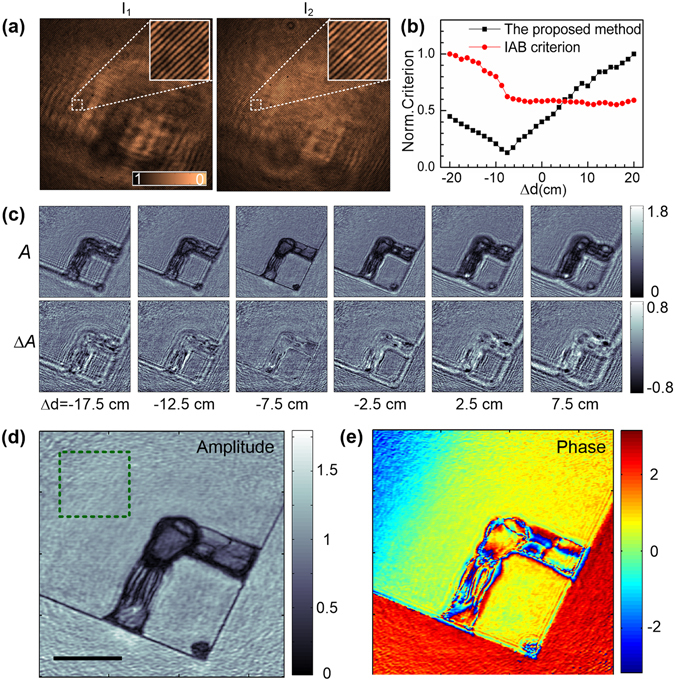
Autofocusing of lensless OV-DHM for a sample with both amplitude and phase modulation. (**a**) Holograms of the sample recorded by rotating the polarizer P to horizontal and vertical directions. The two insets in (**a**) are the zoomed areas indicated with the two dash-white close-ups in (**a**). (**b**) The focus criterion curves of the proposed method and a conventional focus criterion: intensity analysis based method (IAB method). (**c**) Reconstructed amplitude *A* of *O*_1_ (top) and, the difference Δ*A* between |*O*_1_| and |*O*_2_| (bottom). (**d**) Averaged amplitude and (**e**) phase distributions of *O*_1_ and *O*_2_ reconstructed with Δd = 40 ± 7.5 cm. Scale bar in (**d**), 1 mm. The green close-up in (**d**) indicate the area on which speckle noise level is evaluated for *O*_1_, *O*_2_, and (*O*_1_ + *O*_2_)/2, respectively.

Lensless OV-DHM was firstly performed on the configuration sketched in Fig. 1 omitting the imaging units MO_1_-L_3_ and MO_2_-L_4_. A structured glass plate which has both amplitude and phase modulations was used as a sample. The sample was located in the plane having 7.5 ± 0.1 cm distance from the plane P_mid_, which has an equal distance of 40 cm to the CCD plane clockwise and anti-clockwise. Figure 3(a) shows the two holograms *I*_1_ and *I*_2_ obtained by rotating the polarizer P to horizontal and vertical directions. The two object waves (*O*_r1_ and *O*_r2_) were reconstructed and propagated for a distance of −40 cm ± Δ*d* with Δ*d* varying between −20 cm and 20 cm. For each reconstruction distance, the focus criterion defined with Equation (2) was shown in Fig. 3(b). The proposed criterion shows a minimum at Δ*d* = −7.5 ± 0.2 cm, which is in good agreement with the prior-set value. Here the error 0.2 cm is the calculation step for digital re-focusing. In contrast, the conventional criterion, e.g., intensity analysis based (IAB) criterion, failed to find the correct image plane. This is because the phase distribution of the object wave introduces additional intensity variation in out-of-focus planes, which balances surpass the intensity variation of the object wave in the image plane. In Fig. 3(c), we show the reconstructed |*O*_r1_| and |*O*_r1_| − |*O*_r2_| at different Δ*d*. It is distinct that the variation between |*O*_r1_| and |*O*_r2_| becomes minimal at the image plane. In contrast, the intensity modulation of a single object wave, e.g., *O*_1_, does not appear a monistic change with Δ*d*. This, in turn, explains why the conventional IAB criterion can not find the correct image plane. For a sample with both amplitude and phase modulation, the advantage of the proposed method over the conventional autofocusing methods, *i.e*., integrated amplitude modulus (IAM), Laplace Filtering based differential method (LAP), Variance of intensity distribution (VAR) based methods, has also been verified (see Supplementary Text1 and Supplementary Fig. 3). Eventually, by using the obtained Δ*d* = −7.5 ± 0.2 cm, the focused amplitude and phase image of the sample were reconstructed and shown in Fig. 3(d) and (e). A clear area on |*O*_r1_|, |*O*_r2_| and (|*O*_r1_| + |*O*_r2_|)/2, indicated with the green rectangle in Fig. 3(d), were selected for comparison on speckle noise. The standard deviations on their intensity fluctuation 0.30, 0.36 and 0.21 were obtained, implies that speckle noise can be efficiently reduced by averaging the two reconstructed object waves.

### Autofocusing of lens-based OV-DHM

**Figure 4:**
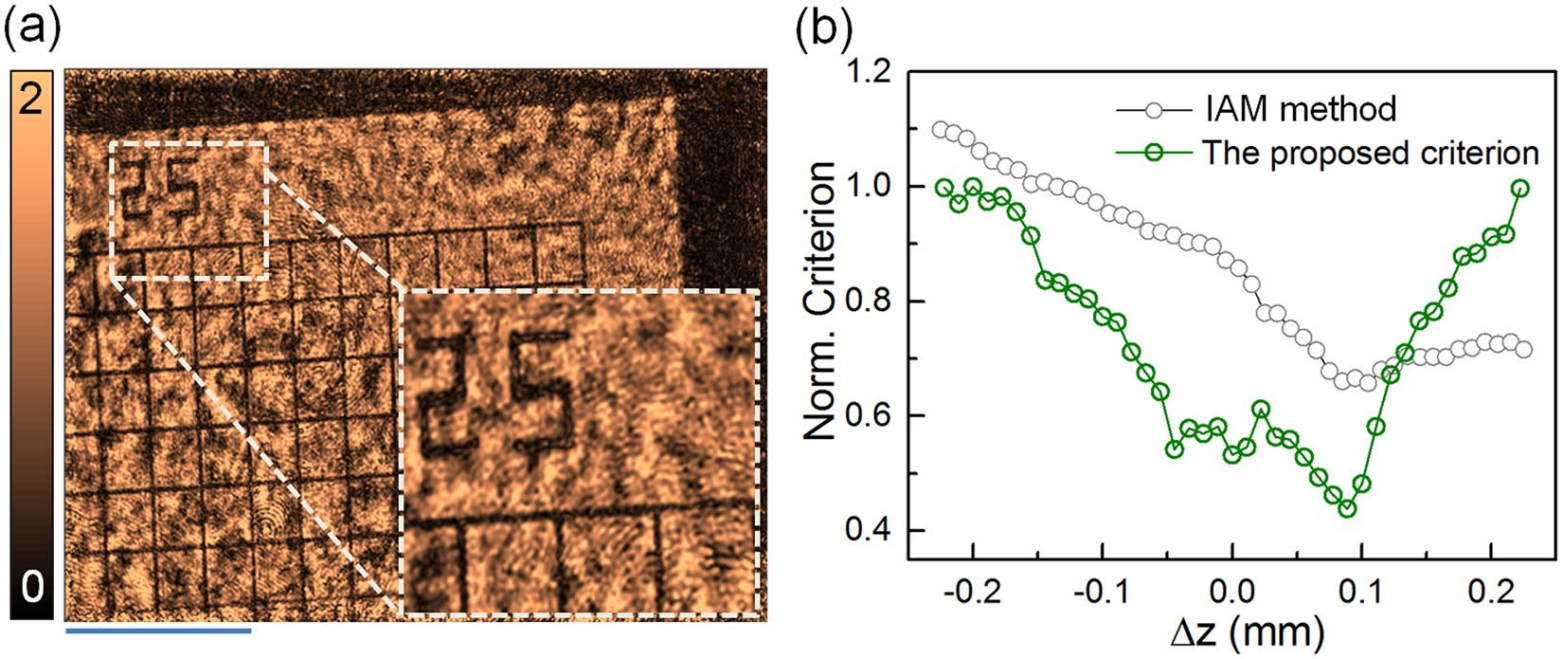
OV-DHM for amplitude sample. (**a**) Reconstructed amplitude of the sample; scale bar 75 μm; the inset in (**a**) shows the zoomed image of the rectangular area indicated with the dash square. (**b**) Focus criterion curves calculated with the proposed method and the integrated amplitude modulus based method, for which the integral of the amplitude modulus is minimized when the focused plane is reached^6^.

**Figure 5:**
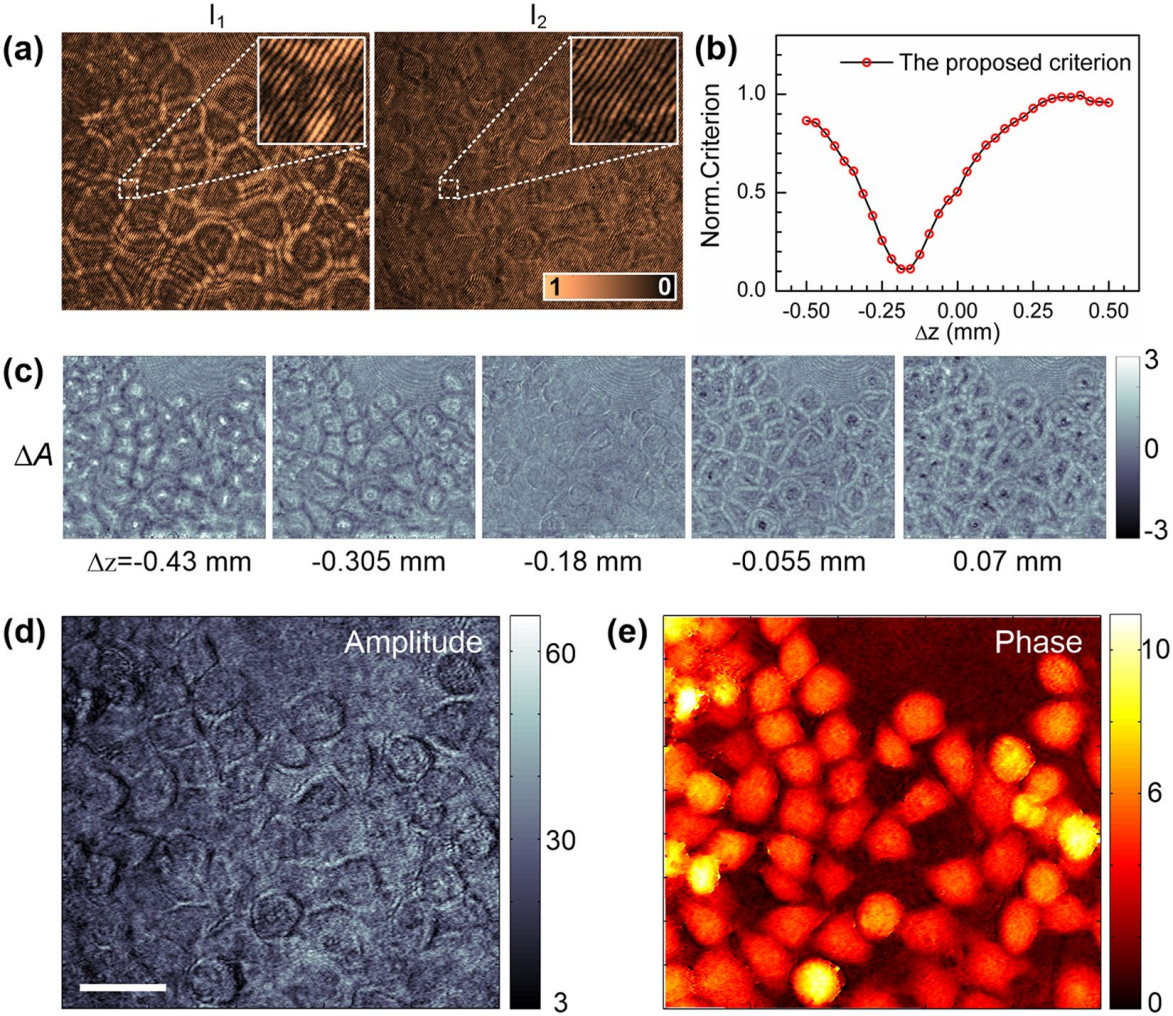
OV-DHM for Hela cells. (**a**) Two opposite-view holograms of the two object waves *O*_1_ and *O*_2_. Insets show the zoomed rectangular areas within the white close-ups in (**a**). (**b**) Focus criterion curves calculated with the proposed method. (**c**) The difference between the amplitudes of |*O*_1_| and |*O*_2_|. (**d**) Reconstructed amplitude and (**e**) phase of the object wave *O*_1_. Scale bar in (**d**), 45 μm.

Lens-based OV-DHM was firstly carried out on a rectangular-grid target (R155731-09100, Edmund Optics, Barrington, NJ, USA) with sharp absorbing structures, as is shown in Fig. 4(a). After the two opposite-viewed holograms were recorded, the focus criterion of the proposed method was calculated and compared with the focus criterion (IAM)^7^ in Fig. 4(b). The IAM method determined the image plane at Δz = 95 μm with which the amplitude modulus is minimized for an amplitude object. The proposed criterion tells the focus plane at Δz = 89 μm. The deviation between the two methods 6 μm is within the range of the depth of field (DOF) of the imaging system (2λ/NA^2  ^=7μm). To follow up, the lens-based OV-DHM was carried out for microscopic biological sample. Human HeLa cells (LGC Standards GmbH, Wesel, Germany) sat on a coverslip surface and covered with another coverslip was used as a microscopic sample. Figure 5(a) shows the two opposite-view holograms *I*_1_ and *I*_2_, and the zoomed areas of *I*_1_ and *I*_2_ highlight the dense fringes due to the angle between the object wave and the reference wave. After calculated with Equation (2), the focus criterion in Fig. 5(b) shows a minimum at Δ*z* = −180 ± 5 μm, which was further verified by Fig. 5(c) where the amplitude difference *O*_r1_ and *O*_r2_ reaches its minimum at Δ*z* = −180 μm. By using the obtained Δ*z*, the amplitude and phase images of the HeLa cells were reconstructed and shown in Fig. 5(d) and (e), respectively. The comparison between Fig. 5(d) and (e) reveals that phase image of these HeLa cells has higher contrast compared with their amplitude/intensity image, which is available with a conventional microscope. We note that the refractive index of the cell can be further determined by using the method proposed in refs 31 and 32.

### Field Of View (FOV) extension by OV-DHM

**Figure 6:**
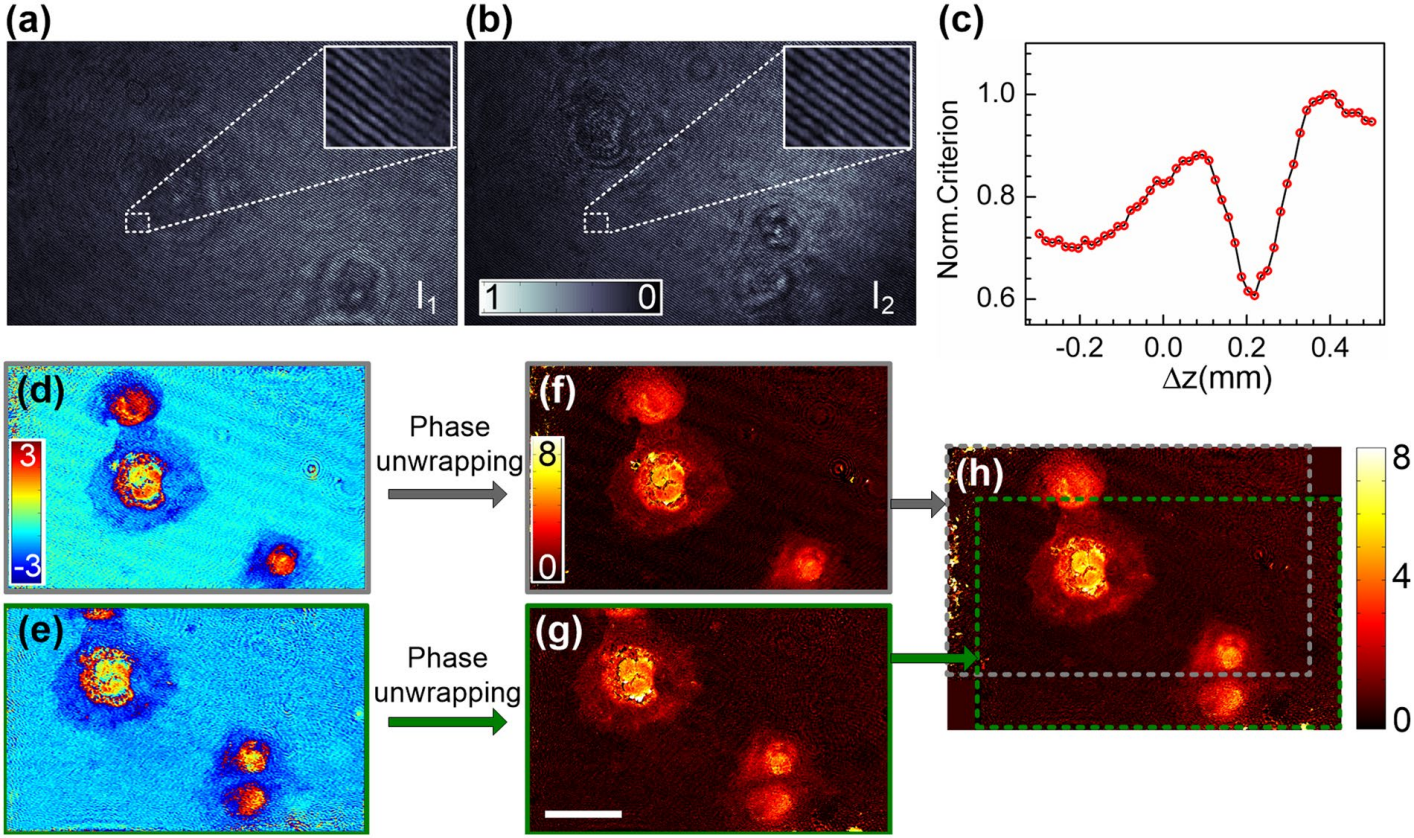
Extension of field of view (FOV) by OV-DHM. (**a**) and (**b**) opposite-view holograms of Cos7 cells (Sigma-Aldrich, St. Louis, MO); (**c**) criterion for image plane determination; (**d**) and (**e**) wrapped phase of O1 and O2; (**f**) and (**g**) unwrapped phases of O1 and O2; by using the Goldstein phase-unwrapping algorithm;^32^ (**h**) combined phase image for field of view (FOV) extension. The phase value in (**f**)–(**h**) is reversed for a better display contrast. Scale bar in (**g**), 90 μm.

The OV-DHM has also the ability to extend the field of view (FOV) of phase imaging. To achieve this purpose, the two object waves were aligned to have an angle α in-between (after magnified by the objectives). By doing this, the CCD camera recorded different parts of the sample image/diffraction pattern along two object waves (see Fig. 6(a) and (b)). In this case, image plane determination can still be performed by replacing |O_r1_(x,y,Δ*d*)|^2^ − |*O*_r2_(*x*, *y*, −Δ*d*)|^2^ in Equation (2) with |*O*_r1_(*x*, *y* + [tan(α/2)]Δ*d*, Δ*d*)|^2^ − |*O*_r2_(*x*, *y* − [tan(α/2)]Δ*d*, −Δ*d*)|^2^, supposing the angle between the two object waves is in y direction (the angle can be in any direction). The focus criterion was calculated from the central area where the two opposite-view object waves are overlapped, and the result was shown in Fig. 6(c), from which the reconstruction distance Δ*d* = *M* ^2^Δz = 8.4 ± 0.2 cm was obtained. The phase images of *O*_1_ and *O*_2_ reconstructed with Δ*d* = 8.4 cm were shown in Fig. 6(d) and (e), and the counterparts after phase unwrapping operation^33^ were in Fig. 6(f) and (g). It can be seen that the two phase images exhibit different parts of the tested sample. After the two images were combined together, the whole field of view is extended from 1.2 × 10^5^ μm^2^ to 1.6 × 10^5^ μm^2^ in Fig. 6(h); that said, 30% FOV extension was achieved by the OV-DHM. The combined image shows Cos7 cells proliferation status: one to two after 24 hours relocation on a glass plate. The phase image also confirms that the nucleus is much denser in refractive index than that of cytosol in a cell.

## Discussions

In this paper, opposite-view digital holographic microscopy (OV-DHM) was presented for autofocusing and field of view (FOV) extension. The out-of-focusing distance (a distance between the image plane and CCD plane) is determined by searching the minimal difference between two object waves reconstructed from two opposite-view holograms. Based on this distance, refocusing of the sample has been performed by propagating the reconstructed object waves to their common image plane. Compared with the conventional image plane determination methods, this method does not rely on the type of a sample (i.e., can be used for the sample with both amplitude and phase modulations). This advantage is due to the intrinsic illumination scheme of OV-DHM and is valid for both lensless OV-DHM and lens-based OV-DHM. The autofocusing can be performed on different regions of the sample: by refocusing and resorting different laterally-separated regions of a sample into their correct axial planes, a 3D image of the sample can be obtained (Supplementary text 4 and Supplementary Figs 4–6). Furthermore, averaging the two object waves can also contribute to suppressing the out-of-focusing background, since only the in-focus components (e.g., cells) appear the same in the two images, wherever the out-of-focus components have different defocusing distance in the two images (see Supplementary Figs 5 and 6).

In case there is an angle between the two opposite-view object waves, extension of the field of view (FOV) can be performed by combining the two reconstructed images. Since the lateral shift of the two reconstructed object waves has a linear relation with the defocusing distance,^22^ out-of-focus information and speckle noise can be suppressed by averaging the two object waves (Supplementary Figs 5 and 6). Furthermore, the angle between the two illuminations has also the potential to improve the lateral resolution of the OV-DHM, considering a larger spectrum can be synthesized by combining the spectra of the two off-axis propagated object waves^22–24^. It is worthy to notice that there is a conflict between FOV extension and the improvement on sectioning and resolution improvement, since they rely on different experimental setting. To extend the FOV, the sample should be placed has a certain distance Δz from the middle plane of the two objectives (which corresponds to the in-focus plane). This defocus distance Δz will provide a lateral shift of 2tan(α/2)M^2^Δz between two opposite-view images in CCD/CMOS plane, which is used for FOV extension. Whereas, to improve the resolution and sectioning ability, a sample (or a section of it) should be located in the middle plane of the two objectives, where the two object waves will have the same image on CCD/CMOS plane. The potential of OV-DHM on improving section ability could be further explored by using two coherent opposite-view illuminations to generate a structured illumination with in the axial direction, similar to I^5^M microscope.^34^ Furthermore, the two object waves with orthogonal polarizations have also the potential to provide polarization-resolved information for the anisotropic sample^35^.

The OV-DHM has also the following disadvantages: firstly, the configuration of the OV-DHM is far more complex than that of the convention DHM, and the requirement of two opposite-view object waves makes it not compatible with a common microscope frame. Secondly, the autofocusing of OV-DHM requires symmetry on two opposite-view object waves. Thus, the two waves should be aligned carefully, and collimated well to avoid defocusing and other aberrations.

## Methods

### OV-DHM Setup and alignment

The OV-DHM setup was constructed according to the sketch in Fig. 1. For the lensless OV-DHM mode, the imaging units MO_1_-L_3_ and MO_2_-L_4_ was omitted. The middle plane of the mirror M_1_ and M_2_ has the same distance 40 cm to the CCD plane along clockwise and anti-clockwise directions. For the lens-based OV-DHM mode, two telescope systems MO_1_-L_3_, and MO_2_-L_4_, were used to image samples with a magnification of 20x. *MO*_1_ and *MO*_2_ are two identical, plan-field microscopic objectives (Plan 25X/0.4, Nanjing Yingxing Optical Instrument Company, Nan Jing, China). L_3_ and L_4_ are the achromatic lens (AC254-200-A-ML, Thorlabs GmbH, Munich, Germany) with focal length 200 mm and diameter 1 inch. An additional telescope system with a magnification M = 1.5 was placed before the CMOS camera to further magnify the object waves.

The following procedure can be performed to make the two opposite-view object waves along the same line: A circular aperture (diameter: 2 mm) was located in the center of the input beam before the polarization beamsplitter PBS (PBS251, Thorlabs GmbH, Munich, Germany). Then, adjust the mirror M_2_ to make sure the two circular patterns (of the clockwise-propagated and anti-clockwise-propagated beams) on the mirror M_1_ are overlapped. Similarly, adjust the mirror M_1_ to make sure the two circular patterns on the mirror M_2_ are overlapped. In addition, the angle between the in-line object waves (*O*_1_ and *O*_2_) and the reference wave was set to 10 ± 0.3 mrad, which was evaluated by Fourier analysis on the off-axis hologram^36^. The generated off-axis hologram was recorded by a Complementary metal–oxide–semiconductor (CMOS) camera (1920 ×1200pixels, 5.86 μm/pixel, 54 fps, DMK 23UX174, Imaging Source, Bremen, Germany). In the reconstruction of the hologram, an asymmetric window function *W*_*filter*_(*ξ*,*η*) was used to collect more high-frequency spectrum in the directions other than the carrier-frequency direction (see Supplementary Fig. 2).

Compared to an in-focus recording hologram, the out-of-focus recording hologram requires increased spatial bandwidth product (SBP), and the SBP consumption increases with the out-of-focus recording distance.^37, 38^ We note that this requirement can be released by using a larger magnification in the OV-DHM system or utilizing a Slightly-off-axis^4, 30^ and on-axis^39, 40^ recording scheme. In our experiment, a CCD with pixel size 1/4.4 AU (airy unit, the diameter of the first-order diffraction-limited Airy disc in CCD plane) was used, with which a slight resolution reduction still happen in the case of a large out-of-focus distance. Thus, in order to avoid the SBP deficit and high-frequency cutting by the objective, we limited the out-of-focusing distances in a range of [−*wd*/10, *wd*/10], with the *wd* being the working distance of the used objective.

### Numerical compensation for axial misalignment of OV-DHM

In the lens-based OV-DHM, The two telescope systems were aligned such that the clockwise and anti-clockwise images of the middle plane P_mid_ (of the two objectives MO_1_ and MO_2_) have a defocusing distance of 6 cm and 0 cm on the CCD. For numerical compensation, the reconstruction distance Δ*d* − 0.06 m and −Δ*d* were used (instead of Δ*d* and −Δd) in Equation (2) for image plane determination of *O*_r1_ and *O*_r2_.

### Cell culture and sample preparation

The preparation of the biological samples has followed the protocol in the literature^41^. Human HeLa cells (LGC Standards GmbH, Wesel, Germany) and COS-7 cells (Sigma-Aldrich, St. Louis, MO) were maintained at 37 °C and 5% CO_2_ in Dulbecco’s modified Eagle’s medium (DMEM), containing 10% fetal bovine serum (FBS) and antibiotics (60 µg/mL penicillin and 100 ng/mL streptomycin, both from Invitrogen, Carlsbad, Canada). 24 h after seeding the cells on cover glasses which was placed in the bottom of a plastic-disc container and cultured with the aforesaid medium.

## Electronic supplementary material

Supplementary information

## References

[1] Cuche E, Marquet P, Depeursinge C (2000). Spatial filtering for zero-order and twin-image elimination in digital off-axis holography. Appl. Opt..

[2] Kemper B, Bally GV (2008). Digital holographic microscopy for live cell applications and technical inspection. Appl. Opt..

[3] Osten W (2014). Recent advances in digital holography. Appl. Opt..

[4] Gao P (2011). Parallel two-step phase-shifting digital holographic microscopy based on a grating pair. J. Opt. Soc. Am. A.

[5] Langehanenberg P, Kemper B, Dirksen D, von Bally G (2008). Autofocusing in digital holographic phase contrast microscopy on pure phase objects for live cell imaging. Appl. Opt..

[6] Kemper B (2007). Integral refractive index determination of living suspension cells by multifocus digital holographic phase contrast microscopy. J. Biomed. Opt..

[7] Dubois F, Schockaert C, Callens N, Yourassowsky C (2006). Focus plane detection criteria in digital holography microscopy by amplitude analysis. Opt. Express.

[8] Antkowiak M, Callens N, Yourassowsky C, Dubois F (2008). Extended focused imaging of a microparticle field with digital holographic microscopy. Opt. Lett..

[9] Memmolo P (2011). Automatic focusing in digital holography and its application to stretched holograms. Opt. Lett..

[10] Yu L, Cai L (2001). Iterative algorithm with a constraint condition for numerical reconstruction of a three dimensional object from its hologram. J. Opt. Soc. Am. A.

[11] Gillespie J, King RA (1989). The use of self-entropy as a focus measure in digital holography. Pattern Recognit. Lett.

[12] Ma LH, Wang H, Li Y, Jin HZ (2004). Numerical reconstruction of digital holograms for three-dimensional shape measurement. J. Opt. A: Pure Appl. Opt..

[13] Li W, Loomis NC, Hu Q, Davis CS (2007). Focus detection from digital in-line holograms based on spectral l1 norms. J. Opt. Soc. Am. A.

[14] Liebling M, Unser M (2004). Autofocus for digital Fresnel Holograms by use of a Fresnelet-Sparsity Criterion. J. Opt. Soc. Am. A.

[15] Lee S, Lee JY, Yang W, Kim DY (2009). Autofocusing and edge detection schemes in cell volume measurements with quantitative phase microscopy. Opt. Express.

[16] Tachiki ML, Itoh M, Yatagai T (2008). Simultaneous depth determination of multiple objects by focus analysis in digital holography. Appl. Opt..

[17] Lebrun D, Benkouider A, Coetmellec S, Malek M (2003). Particle field digital holographic reconstruction in arbitrary tilted planes. Opt. Express.

[18] Park Y, Popescu G, Badizadegan K, Dasari RR, Feld MS (2007). Fresnel particle tracing in three dimensions using diffraction phase microscopy. Opt. Lett..

[19] Sheng J, Malkiel E, Katz J (2006). Digital holographic microscope for measuring three-dimensional particle distributions and motions. Appl. Opt..

[20] Javidi B, Yeom S, Moon I, Daneshpanah M (2006). Real-time automated 3D sensing, detection, and recognition of dynamic biological micro-organic events. Opt. Express.

[21] Gao P (2012). Autofocusing based on wavelength dependence of diffraction in two-wavelength digital holographic microscopy. Opt. Lett..

[22] Gao P (2012). Autofocusing of digital holographic microscopy based on off-axis illuminations. Opt. Lett..

[23] Gao P, Pedrini G, Osten W (2013). Structured illumination for resolution enhancement and autofocusing in digital holographic microscopy. Opt. Lett..

[24] Yuan CJ, Situ G, Pedrini G, Ma J, Osten W (2011). Resolution improvement in digital holography by angular and polarization multiplexing. Appl. Opt..

[25] Almoro, P. F. & Hanson, S. G. Object wave reconstruction by speckle illumination and phase retrieval. *J. Eur. Opt. Soc*. **4**, 09002-1-7 (2009).

[26] Almoro P, Pedrini G, Osten W (2007). Aperture synthesis in phase retrieval using a volume-speckle field. Opt. Lett..

[27] Faridian A, Pedrini G, Osten W (2014). Opposed-view dark-field digital holographic microscopy. Biomed. Opt. Express.

[28] Faridian A, Pedrini G, Osten W (2013). High-contrast multilayer imaging of biological organisms through dark-field digital refocusing. J. Biomed. Opt..

[29] Zheng J (2012). Fluorescence volume imaging with an axicon: simulation study based on scalar diffraction method. Appl. Opt..

[30] Shaked NT, Zhu Y, Rinehart MT, Wax A (2009). Two-step-only phase-shifting interferometry with optimized detector bandwidth for microscopy of live cells. Opt. Express.

[31] Kemper B (2006). Investigation of living pancreas tumor cells by digital holographic microscopy. J. Biomed. Opt..

[32] Curl CL (2005). Refractive index measurement in viable cells using quantitative phase-amplitude microscopy and confocal microscopy. Cytometry A.

[33] Goldstein RM, Zebken HA, Werner CL (1988). Satellite radar interferometry: Two-dimensional phase unwrapping. Radio Sci..

[34] Gustafsson MGL, Agard DA, Sedat JW (1999). I5M: 3D widefield light microscopy with better than 100 nm axial resolution. J. Microsc..

[35] Hristu R, Stanciu SG, Tranca DE, Stanciu GA (2016). Improved quantification of collagen anisotropy with polarization-resolved second harmonic generation microscopy. J. Biophoton.

[36] Min JW (2017). Simple and fast spectral domain algorithm for quantitative phase imaging of living cells with digital holographic microscopy. Opt. Letts..

[37] Stern A, Javidi B (2008). Space-bandwidth conditions for efficient phase-shifting digital holographic microscopy. J. Opt. Soc. Am. A.

[38] Claus D, Iliescu D (2013). Optical parameters and space-bandwidth product optimization in digital holographic microscopy. Appl. Opt..

[39] Yamaguchi I, Zhang T (1997). Phase-shifting digital holography. Opt. Lett..

[40] Gao P (2009). Phase-shift extraction for generalized phase-shifting interferometry. Opt. Lett..

[41] Gao P, Prunsche B, Zhou L, Nienhaus K, Nienhaus GU (2017). Background suppression in fluorescence nanoscopy with stimulated emission double depletion. Nat. Photon.

