# Supplementary material for: Opposite-view digital holographic microscopy with autofocusing capability
Source: Sci Rep. 2017 Jun 26;7:4255. doi: 10.1038/s41598-017-04568-x (PMC5484697; doi:10.1038/s41598-017-04568-x)
Supplement: Supplementary file 1 — Supplementary information [file 41598_2017_4568_MOESM1_ESM.pdf]

Supplementary Information:

Opposite-view digital holographic microscopy with autofocusing capability

Juanjuan Zheng[1], Peng Gao[2], and Xiaopeng Shao[1]

[1]*School of Physics and Optoelectronic Engineering, Xidian University, Xi'an, 710071, China*

[2]*Institute of Applied Physics, Karlsruhe Institute of Technology, 76128 Karlsruhe, Germany*

Supplementary Text 1	Simulation: autofocusing of OV-DHM on a thin sample
Supplementary Text 2	Simulation: autofocusing of OV-DHM on a thick sample
Supplementary Text 3	Experimental demonstration: Autofocusing of lensless OV-DHM on a thick sample.
Supplementary Text 4	Multi-focus imaging of lens-based OV-DHM on Hela cells
Supplementary Figure 1	OV-DHM setup with two CCDs
Supplementary Figure 2	Spectrum selection in the reconstruction of OV-DHM
Supplementary Figure 3	Comparison of the proposed algorithm with other autofocusing algorithms.
Supplementary Figure 4	Simulation of OV-DHM multi-focus imaging on a thick sample
Supplementary Figure 5	Multi-focus imaging of lensless OV-DHM on a structured glass plate
Supplementary Figure 6	Multi-focus imaging of lens-based OV-DHM on Hela cells immobilized in argyrols-water jelly (3%, weight/weight)

Supplementary Text

1. Simulation: autofocusing of OV-DHM on a thin sample. A simulation has been carried out to demonstrate the autofocusing capability of OV-DHM. The simulation was based on the OV-DHM configuration shown in Fig.1 in the main text. The magnification of the telescope systems MO_1-L_3 and MO_2-L_4 were set to 10×. The two telescope systems share the same focus plane (P_{mid}), which is imaged on CCD. A specimen was simulated to have the both amplitude and phase distributions[1], as is shown in Supplementary 3(b). The sample was simulated to have a defocus distance Δz=80 μm along clockwise direction. The complex amplitudes of the object waves $O_1(x, y)$ and $O_2(x, y)$ on CCD plane were simulated by numerically propagating the transmitting object wave (through the sample) for a distance of ±Δz [1, 2]. After being magnified by a factor of M=10, the object waves O_1 and O_2 interfere with two reference waves (R_1 and R_2) and resultantly, two holograms were obtained by $I_i=|O_i+R|^2$ with i being 1 and 2, respectively. The object wave O_{r1} (O_{r2}) in the plane, which has a distance $M^2\Delta z$ ($-M^2\Delta z$) from the CCD plane, was reconstructed by using Eq. (1). For each Δz varying from -300 μm to 300 μm, the focus criterion in Eq. (2) was calculated. For each Δz, the amplitude distribution of O_1 and the difference the amplitudes of O_1 and O_2, $|O_{r1}(x, y, \Delta d)|-|O_{r2}(x, y, -\Delta d)|$ is shown in Supplementary 3(a). It is seen that the variation of $|O_{r1}(x, y, \Delta d)|-|O_{r2}(x, y, -\Delta d)|$ is invisible at Δz=-80 μm, and the variation is obvious anywhere else. In the meantime, the criterion curve of the proposed method reaches its minimal at Δz=-80 μm, which agrees with the preset defocus value. In Supplementary 3(c), the proposed method is compared with the conventional autofocusing methods, such as intensity analysis based (IAB) criterion[3], the Laplace operator based differential method (LAP) [4, 5], and the intensity variation based method (VAR)[5, 6]. It can be conclude from the comparison that: for the sample which has both amplitude and phase distribution in space[3-6], the proposed method can find the correct image plane, while the conventional criterions failed to find the correct image plane.

This is due to that phase distribution of an object wave can introduce additional intensity variation in out-of-focus planes, which can balance the real intensity variation of the object wave in its image plane.

2. Simulation: autofocusing of OV-DHM on a thick sample. In this section, multi-focus imaging of OV-DHM is demonstrated for a thick sample. A multi-layer sample, which is comprised of 6 slices and an increment of 0.3mm in-between, was simulated. On each slice, 8 particles, which has both absorption and refraction on an incidence beam, are randomly distributed. Two plane waves were simulated to propagate through the sample slice by slice along two opposite directions. The generated object wave O_1 and O_2 interfered with a common reference waves R, the generated holograms I_1 and I_2 on CCD plane are shown in Supplementary Fig. 4(a) and 4(b), respectively. The two object waves $O_{r1}(x, y, \Delta z)$ and $O_{r2}(x, y, -\Delta z)$ at different axial planes were reconstructed, and one amplitude of $O_{r1}(x, y, -75mm)$ was exemplarily shown in Supplementary Fig. 4(c). In order to perform sectioning, on each slice each pixel of the object waves was judged by a 2D mask, which considers the focus criterion $|O_{r1}|^2-|O_{r2}|^2<0.02$ and the intensity criterion $(|O_{r1}|^2+|O_{r2}|^2)/2<|O_r|_{mean}^2$ with $|O_r|_{mean}=(|O_{r1}|+|O_{r2}|)/2$. The second criterion lies on the fact that the particles have absorption on the transmitted lights. The following filtering was performed for each pixel: the pixel keeps the mean value of $O_{r1}(x, y, \Delta d)$ and $O_{r2}(x, y, -\Delta d)$, when the focus criterion on this pixel is satisfied. Otherwise, the pixel is set to 0. After the same procedure was performed for all the axial slices, a 3D image of the sample was obtained and shown in Supplementary Fig. 4(d). After compared with the simulated 3D sample, it is found out that the particles are correctly sorted in their original positions. It is worthy to mention that: the averaging operation (on the two refocused object waves) can also contribute to suppress the background from out-of-focus particles and consequently, it can improve the signal to noise ratio (SNR) of the reconstructed image.

3. Experimental demonstration: Autofocusing of lensless OV-DHM on a thick sample. In this section, the first experiment has been carried out to test the out-of-focus background suppression ability of OV-DHM. Two structured glass plates, which had a distance of 7.5 cm in-between, were used to mimic a thick, multiple-layer sample. The middle of the two glass plate has a distance 40 cm to the CCD plane along both clockwise and anti-clockwise directions. Supplementary Fig. 5(a) and 5(b) show the two opposite-view holograms recorded by CCD when rotating the polarizer P to 0° and 90°. The two holograms were reconstructed for a distance -(40 cm ± Δ*d*) with Δ*d* changing from -20 cm to 20 cm. Supplementary Fig. 5(c) show exemplarily the averaged amplitude image from the two object waves on the plane with Δ*d*=4.0 cm. On this image there are some in-focus structures (indicated by the black rectangle) and also out of focus, blurred particles (e.g., indicated with the red rectangle). For each sub-region on this image, the in-focus plane can be found by calculating the criterion curve with Eq. (2). Supplementary Fig. 5(e) and 5(f) show exemplarily the focus criterion curves for the two sub-regions selected by the black and red rectangles in Supplementary Fig. 5(c). By using the Δ*d*=-3.5 cm obtained from Supplementary Fig. 5(f), the image of the blurred particle (in the red rectangle) is refocused and shown in Supplementary Fig. 5(d). By doing this, each particle can be localized in 3D space and consequently, 3D particle counting can be performed.

4. Multi-focus imaging of lens-based OV-DHM on Hela cells. The experiment was based on the OV-DHM configuration shown in Fig.1 in the main text. The two object waves were adjusted to have a small angle in-between. Human HeLa cells (LGC Standards GmbH, Wesel, Germany) was immobilized in agarose-water gel (3%, weight/weight), of which the refractive index is 1.3482 measured by refractometer. Supplementary Fig. 6(a) and 6(b) show the amplitude and phase images of the sample reconstructed by propagating two opposite-view object waves to the plane with Δz=-45 μm. For comparison, Supplementary Fig. 6(c) show

the phase image of the two opposite-view object waves reconstructed on the plane with Δz=-210 μm. It can be seen from Supplementary Fig. 6(a)-(c) that only the in-focus cells have the same image in the two opposite-view images. Wherever, the out-of-focus cells are different in the opposite-view images and consequently, they can be suppressed by an averaging operation. In order to quantify the ability of OV-DHM to suppress out-of-focus background, a rectangular area in the left and right images of Supplementary Fig. 6(b), as well as the averaged image of the two, were compared in Supplementary Fig. 6(d). The standard deviations in the three images are 0.49, 0.4, 0.21, respectively. The improvement on the out-of-focus background suppression is mainly due to the fact that, only the in-focus information is enhanced (located in the same area) during the averaging operation, whereas the out-of-focus information which has different lateral shifts is averaged out. In addition, Supplementary Fig. 6(e) and Supplementary Fig. 6(f) show the focus criterion curves for the selected region 1 and 2 indicated with the white and green rectangles in (b) and (c).

Supplementary figures:

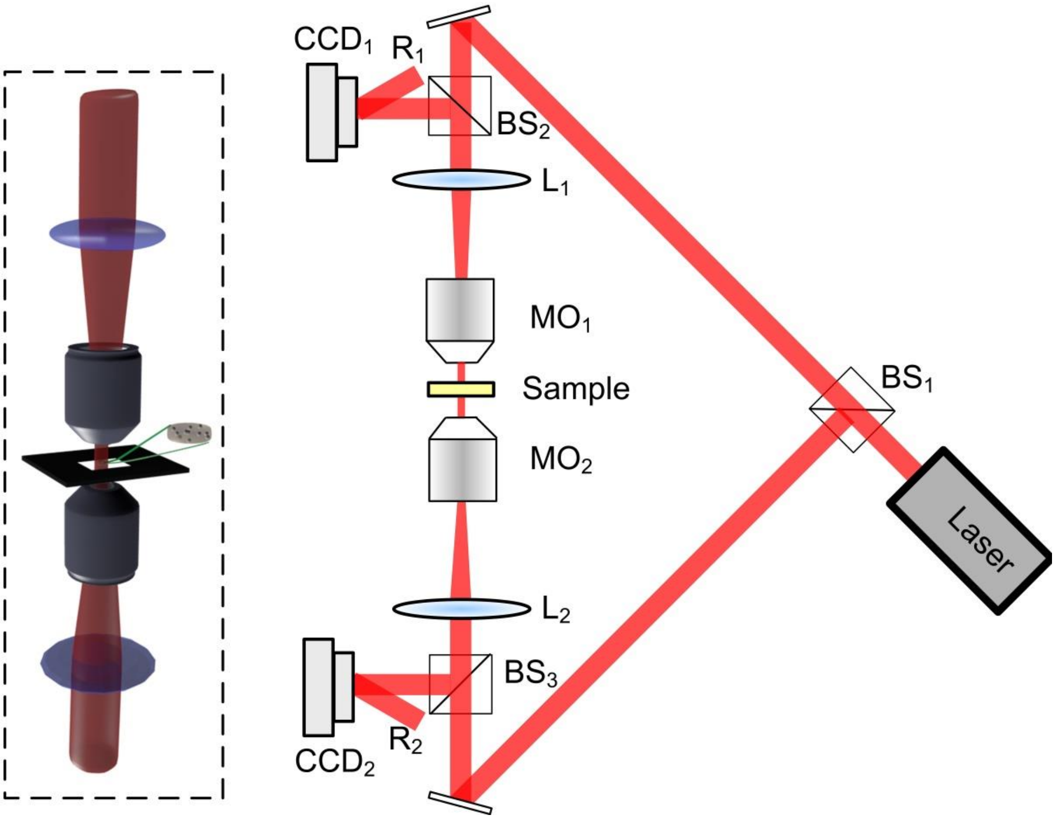

Supplementary Figure S1 | Setup of OV-DHM with two CCDs. MO_1 and MO_2, microscopic objectives; L_1-L_2, achromatic lenses; BS_1-BS_3, beamsplitter; R_1 and R_2, reference waves; P, polarizer; CCD_1 and CCD_2, Charge-coupled device.

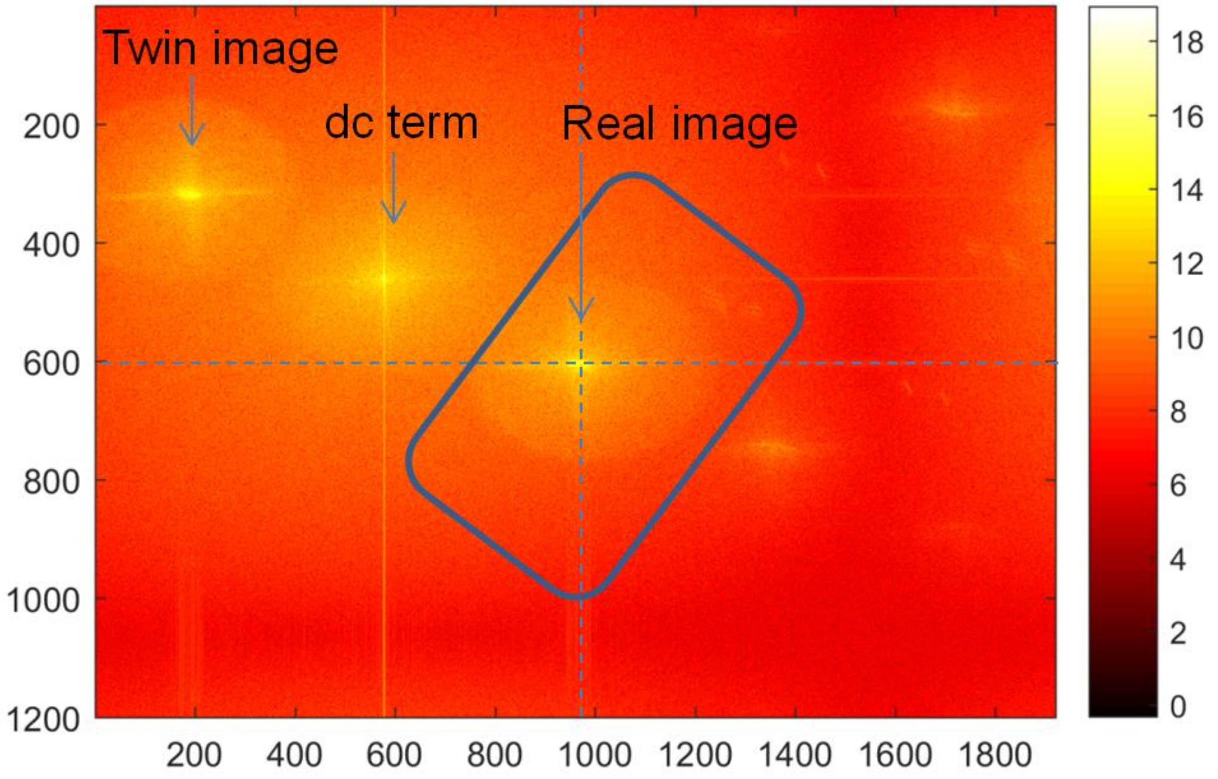

Supplementary Figure S2 | Spectrum selection in the reconstruction of OV-DHM. The area closed up with dash-line indicates the spectrum for the reconstruction of the real image.

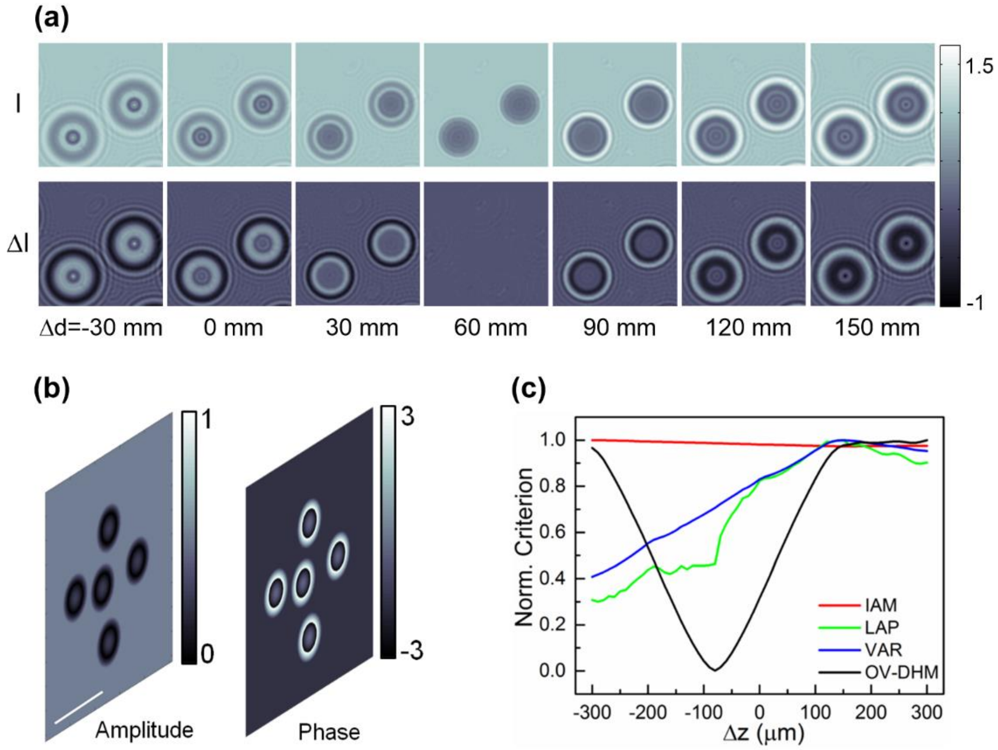

Supplementary Figure S3 | Comparison of the proposed algorithm with other autofocusing algorithms. (a) The amplitude variation of $|O_{r1}(x, y, \Delta d)|-|O_{r2}(x, y, -\Delta d)|$; (b)

Simulated amplitude and phase distribution of an object wave; scale bar, 100 μm. (b) Focus criterion of different methods. The comparison reveals that the proposed method can find the correct image plane for the sample with both amplitude and phase distribution. While, the intensity analysis based (IAB) criterion[3], the Laplace operator based differential method (LAP) [4, 5], and the intensity variation based method (VAR)[5, 6] fail to find the correct image plane for such sample.

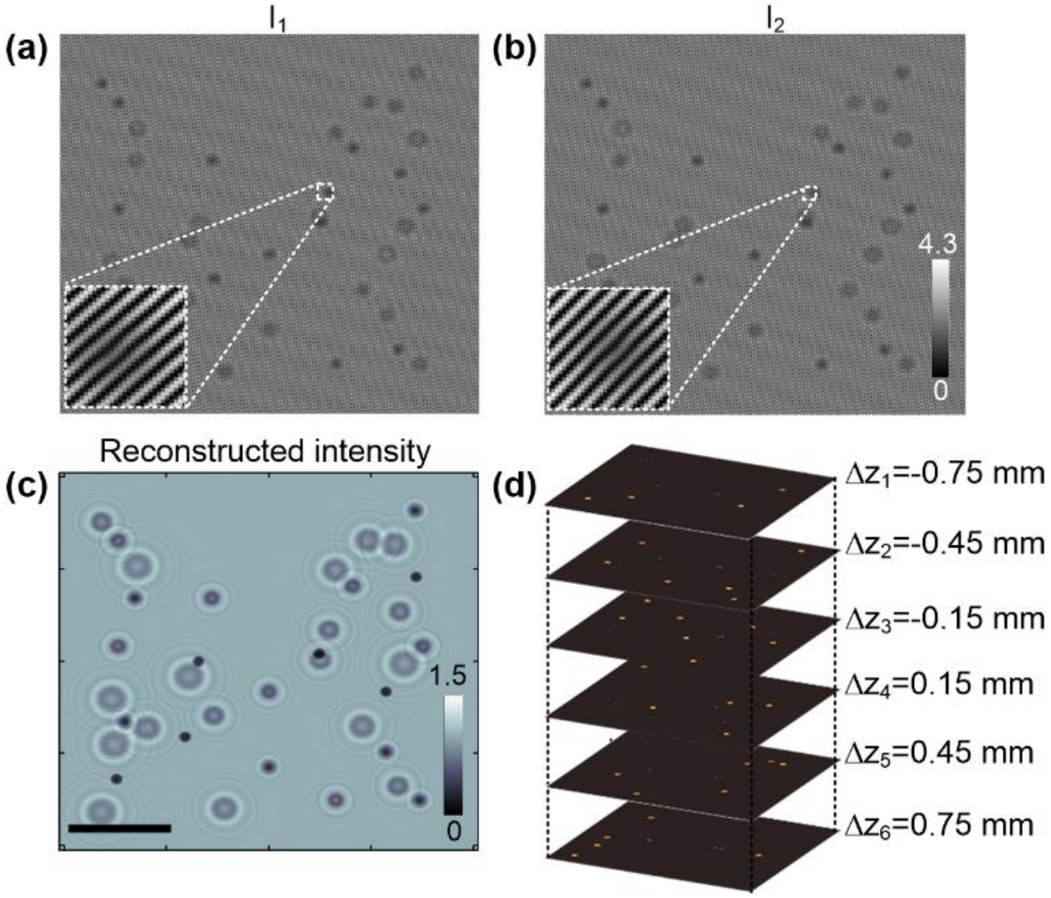

Supplementary Figure S4 | Simulation of OV-DHM imaging on a thick sample; (a) and (b) holograms of the two opposite-view object waves; (c) Amplitude of reconstructed object wave O_{r1} with Δd=-75mm on CCD; (d) reconstructed images in the plane with Δz_1=-0.75mm, Δz_2=-0.45mm, Δz_3=-0.15mm, Δz_4=0.15mm, Δz_5=0.45mm, Δz_6=0.75mm; The gray value in (d) has been inversed in order to enhance contrast of particles. Scale bar in (c), 2 mm.

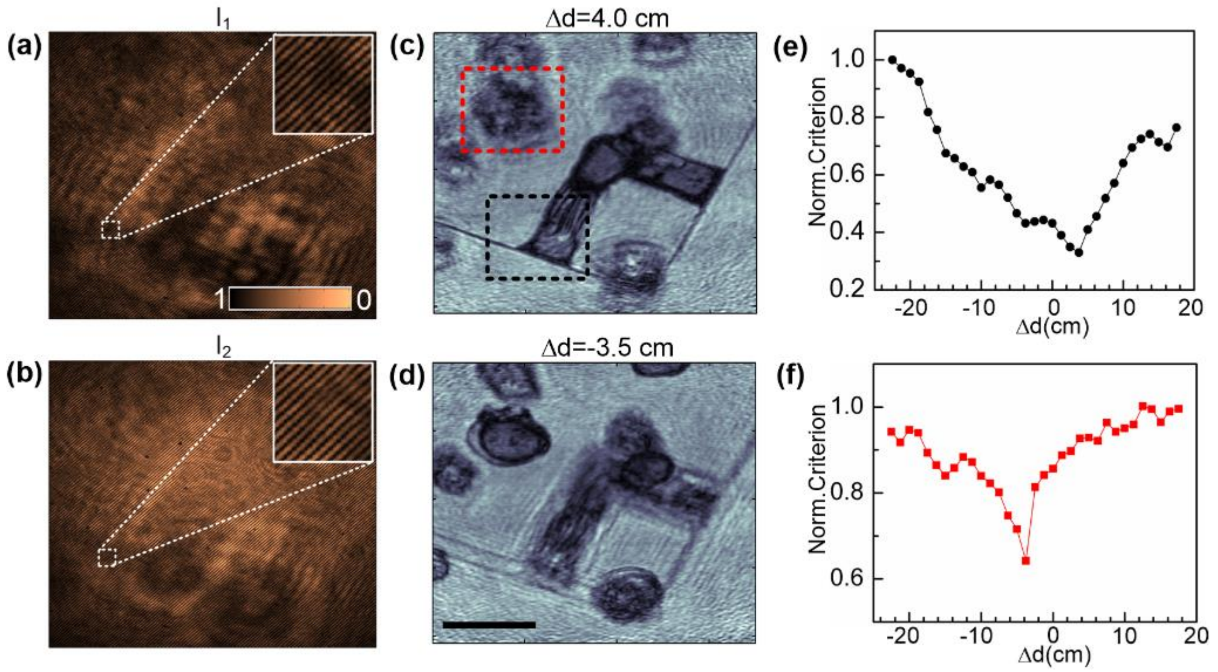

Supplementary Figure S5 | Multi-focus imaging of lensless OV-DHM on a structured glass plate. (a) and (b) Two opposite-view holograms of the two object waves; (c) and (d) the averaged image of the sample of the two object waves on the plane with Δ*d*=4.0 cm and Δ*d*=-3.5 cm. (e) and (f) the focus criterion curves calculated for the two sub-regions indicated with the black and red rectangles in (c) and (d). Scale bar in (d), 1 mm.

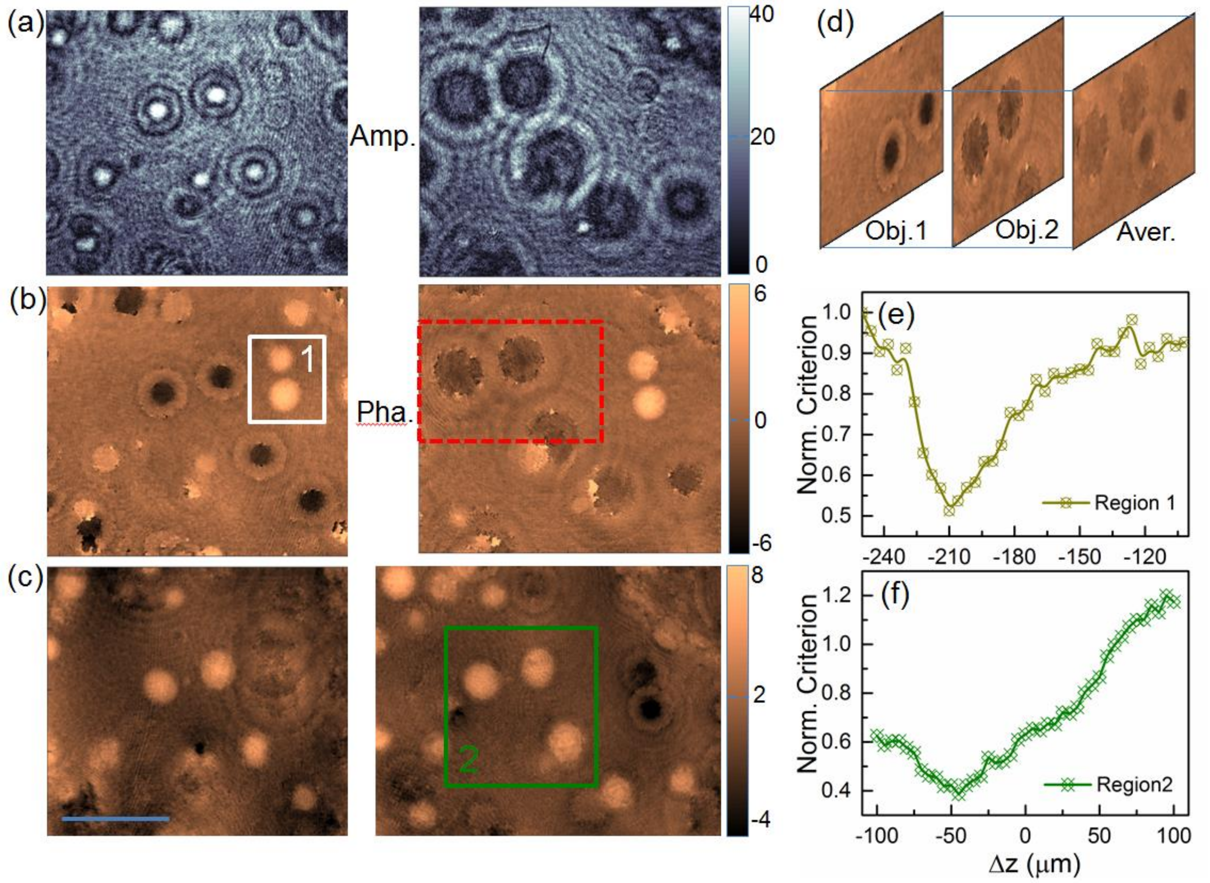

Supplementary Figure S6 | Multi-focus imaging of lens-based OV-DHM on Hela cells immobilized in agarose-water gel (3%, weight/weight). (a) and (b) The amplitude and phase images of the sample on the plane with Δz=-210 μm, which are reconstructed from the two opposite-view object waves (left/right). (c) The reconstructed phase images on the plane with Δz=-45 μm along the two object waves. Scale bar, 40μm. (d) Comparison of out-of-focus background in OV-DHM. Here the images 'Obj.1' and 'Obj.2' are from the same area (indicated by the red rectangle) in the left and right images in (b). The image 'Aver.' was obtained by averaging the two. The standard deviations in the three images in (d) are 0.49, 0.40, 0.21, respectively. (e) and (f) Focus criterion curves for the selected region 1 (the white rectangle) and the region 2 (the green rectangle).

References

1. Zheng, J.J. *et al.* Fluorescence volume imaging with an axicon: simulation study based on scalar diffraction method. *Applied Optics* **51**, 7236-7245 (2012).
2. QUANTA software. School of Crystallography, B.C., University of London, Malet Street, London WC1E 7HX, United Kingdom.
3. Dubois, F., Schockaert, C., Callens, N. & Yourassowsky, C. Focus plane detection criteria in digital holography microscopy by amplitude analysis. *Opt Express* **14**, 5895-5908 (2006).
4. Sun, Y., Duthaler, S. & Nelson, B.J. Autofocusing in computer microscopy: Selecting the optimal focus algorithm. *Microsc Res Techniq* **65**, 139-149 (2004).
5. Groen, F.C.A., Young, I.T. & Ligthart, G. A Comparison of Different Focus Functions for Use in Autofocus Algorithms. *Cytometry* **6**, 81-91 (1985).
6. Ozgen, M.T. & Tuncer, T.E. Object reconstruction from in-line Fresnel holograms without explicit depth focusing. *Opt Eng* **43**, 1300-1310 (2004).
